# Preservation of fertility in female and male prepubertal patients diagnosed with cancer

**DOI:** 10.1007/s10815-023-02945-2

**Published:** 2023-09-29

**Authors:** María Itatí Albamonte, Alfredo D. Vitullo

**Affiliations:** 1 Centro de Estudios Biomédicos Básicos, Aplicados y Desarrollo (CEBBAD), Universidad Maimónides, Hidalgo 775, C1405BCK Buenos Aires, Argentina; 2 Consejo Nacional de Investigaciones Científicas y Técnicas (CONICET), Buenos Aires, Argentina

**Keywords:** Fertility preservation, Cryopreservation, Testis, Ovary, Gametes, Pre- and postpubertal patients, Pediatric cancer

## Abstract

Over the past two decades, the importance of fertility preservation has grown not only in the realm of medical and clinical patient care, but also in the field of basic and applied research in human reproduction. With advancements in cancer treatments resulting in higher rates of patient survival, it is crucial to consider the quality of life post-cure. Therefore, fertility preservation must be taken into account prior to antitumor treatments, as it can significantly impact a patient’s future fertility. For postpubertal patients, gamete cryopreservation is the most commonly employed preservation strategy. However, for prepubertal patients, the situation is more intricate. Presently, ovarian tissue cryopreservation is the standard practice for prepubertal girls, but further scientific evidence is required in several aspects. Testicular tissue cryopreservation, on the other hand, is still experimental for prepubertal boys. The primary aim of this review is to address the strategies available for possible fertility preservation in prepubertal girls and boys, such as ovarian cryopreservation/transplantation, in vitro follicle culture and meiotic maturation, artificial ovary, transplantation of cryopreserved spermatogonia, and cryopreservation/grafting of immature testicular tissue and testicular organoids.

## Introduction

According to estimates provided by the IARC World Cancer Observatory [1], nearly 280,000 children and adolescents between the ages of 0 and 19 were diagnosed globally in 2020, with nearly 110,000 children dying of the disease. Leukemia represents the most frequent oncological disease, followed by central nervous system tumors and lymphomas. Although the actual figures may be higher, as childhood cancer is difficult to diagnose in many countries, early diagnosis, adequate treatment, and comprehensive patient care allow a survival rate between 70 and 80%. These high survival rates suggest that the number of childhood cancer survivors is on the rise, underscoring the importance of considering the quality of life of these patients. This implies not only providing medical follow-up for life, as many survivors suffer serious late effects such as the onset of new cancer, but also the possibility of parenthood in the future.

The reproductive capacity of cancer patients may be impacted by antitumor treatments, particularly when utilizing alkylating agents such as cyclophosphamide, busulfan, and dacarbazine, as noted in previous studies [2–4]. It has been observed that chemotherapy and/or radiotherapy treatment leads to a decline in fertility in roughly 30% of children [4]. The likelihood of gonadotoxicity linked to chemotherapy is dependent upon various factors, such as the specific drugs employed, treatment protocols, dosages, administration intervals, and the age of the patient. Chemotherapeutic medications may be classified as “high or medium risk” (alkylating agents, platinum agents, antitumor antibiotics, and antimetabolites such as cytarabine) or “low risk” (antimetabolites like methotrexate; antitumor antibiotics such as etoposide) with regard to the potential risk they pose to male and female fertility [5]. Consequently, it is imperative to take into account fertility conservation measures prior to commencing treatment. Similarly, it is worth contemplating the inclusion of drugs that are less cytotoxic in the long run, such as vinca alkaloids, which may have an immediate impact on fertility [6].

During the previous two decades, fertility preservation has acquired significance not only in medical and clinical patient care but also in basic and applied research. It encompasses multidisciplinary fields, such as reproductive medicine, oncology, hematology, andrology, toxicology, and psychology, among others, and involves scientific and medical researchers, as well as social workers and nurses. This joint effort enables the enhancement of cancer survivors´ quality of life, as well as individuals with pathologies whose medical treatments endanger their future fertility. Although the oncologist’s and the entire health team’s primary objective is to reduce mortality in cancer patients, their quality of life and potential fertility must also be considered. A survey conducted over two decades by Schover et al. [7] revealed that pregnancy and having a biological child of one’s own are concerns in female cancer survivors. Hence, a comprehensive evaluation and advice to the patient by the treating health team is critical to assess the damage to her fertility and recommend, if necessary, the most appropriate option to preserve it. Currently, several strategies are available for preserving fertility in women and men, whose levels of efficacy are different in each case. This mini-review presents a concise and succinct summary of the current strategies and ongoing experimental procedures (Table 1) aimed at preserving fertility in prepubertal and pubertal girls and boys who are undergoing cancer treatment.

**Table 1:** Fertility preservation strategies

Fertility preservation strategies in the female	
Medical/surgical	-Use of GnRH analogs -Ovarian transposition before radiotherapy
ART	-Embryo -Cryopreservation -Vitrification of human oocytes -Oocyte donation -Ovarian cryopreservation and transplantation -In vitro follicle culture
New research and technologies	-Artificial ovary -Uterus transplantation -Ovarian allotransplantation -Allotransplantation of human ovarian tissue
Fertility preservation strategies in the male	
ART	-Cryopreserved sperm
New research and technologies	-Transplantation of cryopreserved spermatogonia -Cryopreservation and grafting of immature testicular tissue -Testicular organoids

## Ovarian cryopreservation and transplantation

The ovary exhibits a heightened sensitivity towards radiotherapy or chemotherapy treatments. This is particularly noteworthy given the widely held belief in the establishment of a limited and non-regenerative germinal pool of primordial follicles upon birth, which must persist for many decades prior to eventual maturation, growth, and ovulation [8]. Moreover, the germinal reserve of primordial follicles is subject to a continuous elimination, primarily governed by mechanisms associated with programmed cell death, or apoptosis [9–12].

According to the collaborative efforts of the PanCareLIFE Consortium and the International Childhood Cancer Late Effects Guidelines Harmonization Group (IGHG), ovarian tissue cryopreservation (OTC) stands as the sole method currently accessible for the preservation of fertility among prepubertal and peri-pubertal girls, as well as postpubertal women who are not eligible for oocyte cryopreservation [13]. The American Society for Reproductive Medicine (ASRM) ceased the classification of OTC as an experimental practice for pubertal patients in 2019 [14], as a result of the accumulation of evidence regarding the safety of obtaining, freezing, and subsequently performing orthotopic transplantation of ovarian tissue. Additionally, the American Society of Pediatrics (AAP), in collaboration with the Practice Committee of the American Society for Reproductive Medicine [14], advocates for fertility preservation in pediatric patients prior to the commencement of treatment with gonadotoxic agents.

The process of OTC involves the retrieval of ovarian cortical tissue through laparoscopy or laparotomy before administration of gonadotoxic agents. The PanCareLIFE Consortium and the IGHG concur that the technical hazards associated with ovarian tissue resection are minimal and are similar to those associated with any laparoscopic technique, such as infection, bleeding, bowel, bladder, or blood vessel perforation, as well as anesthesia. Additionally, the risks are mitigated by the fact that ovarian tissue resection can be performed concurrently with other surgical procedures [13]. Upon obtaining the tissue, it is fragmented into smaller pieces and subjected to cryopreservation. Among the techniques available, slow freezing and vitrification, the former approach, followed by rapid thawing, has garnered widespread usage and has shown to yield the highest number of live births. This technique has exhibited a survival rate of approximately two-thirds of immature follicles, most of which maintain their morphological normality. Notwithstanding, electron microscopy observations have revealed damage in mitochondria, cell membranes, and the formation of cytoplasmic vacuoles, thereby highlighting the imperfection of current methods and their need for further optimization to minimize the loss of follicles and ovarian function [15, 16]. Vitrification constitutes another cryopreservation technique that involves the use of high concentrations of cryoprotectants to achieve ultra-fast freezing. This method effectively prevents the formation of ice crystals both inside and outside the cell, thereby mitigating the risk of mechanical damage and morphological alteration. Shi and colleagues [17] conducted a meta-analysis to compare the efficacy of ovarian tissue vitrification and slow freezing. Although both techniques yielded comparable outcomes with respect to primordial follicle density and morphology, vitrification exhibited superiority in decreased DNA damage and enhanced preservation of ovarian stromal cells. Nonetheless, one of the primary constraints of vitrification is the use of high concentrations of cryoprotectants, necessitating a brief exposure time (equilibration time) to avoid cell toxicity. The precise calculation of time is crucial in the context of cryopreservation, particularly in relation to tissue penetration by cryoprotectants, as this process differs from that of individual cells. Currently, there are ongoing investigations into the effectiveness of incorporating antifreeze proteins to impede ice-nucleating events, as well as reducing the required dosages of cryoprotectants [18]. Although no universally accepted protocols have yet been established for vitrification of ovarian tissue, studies conducted on bovine specimens have demonstrated comparable efficacy between vitrification using 5.5 M ethylene glycol for 20 min at room temperature and slow freezing [19]. In the present context, the two-step equilibration method utilizing 20% dimethyl sulfoxide and 20% ethylene glycol as colligative cryoprotective agents has been adopted by Donnez’s group for the vitrification of ovarian tissue [5]. Despite the accumulation of information pertaining to cellular damage incurred during the freezing/thawing process, a pressing need exists for further investigation into basic scientific principles, particularly at the molecular level, to facilitate the development of stable, standardized, and optimal tissue cryopreservation protocols for clinical application.

Although fertility preservation for prepubertal girls presents ethical complexities due to the paucity of evidence on the efficacy of OTC for this age group, tissue harvesting is generally considered to be ethically justifiable, as the benefits are thought to outweigh the potential harm in this population, which is at high risk for infertility [13]. It should be noted, however, that ovarian cortex grafts have a short half-life, as approximately two-thirds of the follicular reserve is lost due to ischemic damage after transplantation. The first live birth from OTC of a 25-year-old woman with grade IV Hodgkin lymphoma and subsequent orthotopic autotransplantation was reported in 2004 [20]. Since then, there has been an exponential increase of pregnancies and live births, with over 200 recorded to date [21]. Orthotopic transplantation of cryopreserved and thawed ovarian tissue has been the most successful method thus far. Worldwide live birth rates from OTC in adult patients have been reported as 31% in the Danish group in 2015, 25% in the German FertiPROTEKT network in 2016, 18.2% in the Spanish group in 2018, and 41.6% in a Belgian-Israeli-American case series reported in 2020 [22–25]. In 2021, a study involving 285 patients across five leading European centers reported an overall pregnancy rate of 38% and a live birth rate of 26% [26]. As for heterotopic transplantation, only one live birth has been reported in humans [27].

With respect to the prepubertal cohort, a spontaneous pregnancy resulting in live birth was reported in 2015, following autotransplantation of thawed ovarian tissue from a female who had undergone OTC prior to menarche. Additionally, it was observed that ovarian function resumed between 60 and 240 days post transplantation and persisted for up to 7 years. As the efficacy of autotransplanted ovarian tissue in maintaining endocrine function over the long-term is low, it is recommended to perform the procedure when the patient is preparing for conception [28]. After the favorable outcome, an additional 15 patients were documented. Of these, nine were diagnosed with a malignant ailment, whereas the remaining six were not. Conversely, five of the patients had not yet undergone menarche prior to OTC treatment, while eight had already undergone chemotherapy. Following ovarian tissue removal, all patients underwent gonadotoxic treatment. In 80% of the patients, ovarian function resumed, including 3 girls who were prepubertal at the time of OTC. Furthermore, of the 15 patients, 9 conceived at least once (60%) and 7 gave birth to at least one child (47%), including 2 who were not pubertal at the time of OTC [28–32]. Importantly, of the 15 patients, 9 conceived at least once (60%) and 7 delivered at least one child (47%), including 2 who were prepubertal at the time of OTC [28–32].

The PanCareLIFE Consortium and the IGHG have deemed autotransplantation as the sole means of cryopreserving ovarian tissue for fertility restoration that is appropriate for postpubertal patients. However, they recommend a careful evaluation within the framework of a clinical trial. On the other hand, for the prepubertal patients, the transplantation of cryopreserved ovarian tissue must solely be proposed only in the context of an experimental procedure [13].

## In vitro follicle culture and meiotic maturation

The potential risk of reintroduction of malignant cells during autotransplantation of cryopreserved ovarian tissue, particularly in leukemia, non-Hodgkin lymphoma, and metastatic solid tumor survivors, has been established by several studies [13, 33, 34]. Various techniques have been employed to detect disseminated tumor cells in cryopreserved tissue before transplantation. These methods include standard histopathological analysis with hematoxylin-eosin and immunohistochemistry and molecular analysis for the detection of chromosomal abnormalities (RT–PCR amplification), flow cytometry, fluorescence in situ hybridization, and xenografting to immunodeficient mice [5]. Currently, novel and more secure methodologies are being developed for the acquisition of oocytes suitable for assisted reproductive technology (ART), such as in vitro follicular growth. This technique is complex as it must replicate all the stages of activation and follicular development that occur in vivo. The female reproductive function necessitates the cyclical and maturational development of ovarian follicles, which originates from the continuous activation of the primordial follicle mass. The process of follicular development involves a sequence of controlled events, which are characterized by transition stages that start with the initiation of growth of primordial follicles to the secondary follicle stage, formation of antral follicles leading to the De Graaf follicle stage, involving the association of granulosa cells and accumulation of antral fluid, and culminating in the acquisition of a mature oocyte. Hence, to ensure the adequate follicular growth and oocyte maturation to occur in vitro, a multiple-stage culture system must be implemented to provide the necessary requirements for each developmental stage [35–37]. A four-stage culture system is currently proposed. The first stage involves the activation and initial growth of the primordial follicles up to the secondary follicle stage, followed by the second stage which entails growing secondary follicles to the antral follicle stage. The third stage involves complete oocyte growth, and the final fourth stage encompasses oocyte maturation in vitro [38]. Successfully carrying out the first stage of cultivation is noted to be one of the most complex processes. This is because the quiescent primordial follicle reserve in ovarian cortex fragments must initiate an activation process that allows follicle growth. Although not well understood in humans, primordial follicle activation is a necessary step to develop an optimal follicular growth system in vitro. Several culture systems that support the activation of human primordial follicles have been developed [35, 37–45]. However, the variable outcomes observed suggest the significance of the individual cellular components and the signaling pathways that regulate follicular activation [46]. Among these pathways, the phosphatidylinositol-3′-kinase (PI3K-AKT) path has been studied in knockout mouse models [47] and in human ovarian cortex cultured in vitro [48–50] and plays a crucial role in this process. Growth factors such as follicle stimulating hormone (FSH) stimulate PI3K, which activates a phosphoinositide-dependent protein kinase-1 (PDK1). This, in turn, phosphorylates Akt and downstream transcription factors, such as FOXO1 (forkhead winged helix box O1) and FOXO3 (forkhead winged helix box O3), leading to follicular activation and growth [51, 52]. Conversely, deletion of the *PTEN* (*phosphatase and tensin homolog*) gene has been found to stimulate the growth of primordial follicles in neonatal and adult animals [47, 53, 54]. This gene encodes a phosphatase that is responsible for negatively regulating the PI3K-AKT signaling pathway. It has been observed that the deletion of *PTEN* leads to an increase in AKT phosphorylation and nuclear export of the transcription factor FOXO3 (forkhead box O3) [53, 55, 56]. In addition, it has been observed that the effects of PTEN can be inhibited pharmacologically in a reversible manner by vanadate (bisperoxovanadium) derivatives that act as tyrosine phosphatase inhibitory proteins, thereby promoting downstream AKT phosphorylation and stimulating in vitro activation and growth of primordial follicles [48, 57–59]. Despite the fact that primordial follicle activation increases, the quality of secondary follicles is poor due to DNA damage and insufficient DNA repair mechanisms [60]. Information on the expression of PTEN and FOXO3 in human ovaries is sparse. The examination of *PTEN* and *FOXO3* of different developmental stages in human ovaries revealed the presence of two distinct populations of primordial follicles during the postnatal period, one expressing nuclear *FOXO3* and the other not [61]. Conversely, in mice, all primordial follicles express nuclear FOXO3, which translocates to the cytoplasm during activation using an “all or nothing” mechanism. It is possible that FOXO3-expressing primordial follicles in humans follow a similar pattern to ensure long-term fertility. However, the dynamics of FOXO3 expression among non-FOXO3- and FOXO3-expressing primordial follicle populations in humans require further elucidation. Another player involved in the PI3K-AKT pathway is mTORC1 (mammalian target of rapamycin complex 1), a serine/threonine kinase that regulates cell growth and proliferation in response to growth factors and nutrients, which plays a role in the activation of primordial follicles [62]. As demonstrated in mTORC1 knockout mice, the activation of primordial follicles is increased [63]. Furthermore, the Hippo pathway, which regulates organ size by controlling cell proliferation and death processes [64], also contributes to primordial follicle activation. In vitro interruption of the Hippo pathway causes the activation of primordial follicles in cortices of ovarian tissue [50, 65].

Once primordial follicles in the ovarian cortex fragment are activated, the second culture stage involves mechanical and/or enzymatic removal of multilaminar follicles. The mechanical removal allows for the preservation of follicle integrity by conserving the basal lamina and the thecal cell layer. However, this method is laborious and operator dependent, resulting in a low yield [36, 66]. Isolated follicles are individually cultured in the presence of FSH and activin until they mature into antral follicles. Subsequently, the cumulus-oocyte complexes undergo a third stage of culture with activin-A and rhFSH until the oocyte attains a size of around 100 μm [35, 36]. The final stage of culture involves the maturation of the oocytes to the MII stage. Despite showing variable results in humans, in vitro meiotic maturation resulted in the first live birth from an in vitro matured oocyte in 1991 [67–70]. However, Revel et al. [71] reported the ex vivo maturation of oocytes obtained from small antral follicles removed from the ovary of adult patients. Since then, several publications have reported different maturation strategies for oocytes collected from patients spanning between birth and 44 years of age [71].

In conclusion, it must be noted that in vitro follicle culture and meiotic maturation remain experimental procedures, lacking sufficient scientific evidence. Further research is required to deepen our knowledge regarding the activation of the primordial follicles, the maintenance and development of follicles in vitro, the maturation of the oocyte, and their quality to be used in ART. Likewise, it must be considered that prepubertal patients contain five times more abnormal follicles that do not mature compared to postpubertal patients. In addition, it was observed that fewer cultured primordial follicles advance to the secondary stage, and some even contain oocytes that lack the membrane of the germinal vesicle (GV) or the nucleolus [38].

## Artificial ovary

Ovarian follicles, isolated from ovarian tissue fragments and cultivated in a scaffold, are capable of producing a synthetic organ that can be transplanted to either an orthotopic or heterotopic location. The resulting artificial ovary serves both the reproductive and endocrine function, i.e., the production of gametes and the release of steroid hormones, respectively [72–77]. Following the mechanical or enzymatic isolation with collagenase [78], the type and number of follicles that will grow in the scaffold must be determined. Chiti et al. [79] demonstrated that secondary follicles respond better in terms of survival and growth rate than primordial and primary follicles. Additionally, the number of follicles in the scaffold has to be adjusted appropriately to maintain a small size of the delivery matrix [71]. The scaffold design is critical for the adequate growth of the follicles and for the safety of the patient after transplantation. Furthermore, it must allow neoangiogenesis to supply oxygen and nutrients to the cells [80–82]. Since the 1990s, various biomaterials, including collagen, fibrin, alginate, alginate-matrigel, poly (ethylene glycol) vinyl-sulfone (PEG-VS), fibrin-VEGF, fibrin-alginate, fibrin-collagen, plasma clot, and fibrin-hyaluronic acid, have been tested in mice to construct the artificial ovary [81–91]. The last two materials have been tested with human ovarian tissue [86, 92]. Notwithstanding the array of biomaterials that have been examined, a great deal of research remains to be conducted to ascertain the most suitable one for follicular development. Rajabzadeh et al. [90] demonstrated a follicular recovery rate of 48.31% over a period of 14 days following transplantation, utilizing a scaffold composed of fibrin gel and platelet lysate. It is possible to transplant artificial ovaries into orthotopic sites, such as the ovary, pelvic cavity, and peritoneal window, as well as heterotopic sites, like the rectus muscle, forearm, and neck. However, the latter approach does not allow for natural conception and necessitates consideration of factors such as differences in body temperature, pressure, paracrine factors, and blood supply [73, 77, 89]. Immune rejection and ischemic injury must both be taken into account in the transplantation of the artificial ovary. Despite the autologous origin of the ovarian cells, it is vital to consider the biomaterials employed in the scaffold, as these could trigger an immune response; thus, their design is of paramount importance [93–95]. Besides, to prevent ischemic injury, appropriate neovascularization is necessary after transplantation [20, 96, 97].

Although there is still much ambiguity surrounding the most effective approach to developing an artificial ovary, it is worth noting that there have been few reported cases of successful pregnancies in animals [81, 89]. However, advancements in tissue and organ engineering have opened up a new avenue for exploration in the pursuit of fertility preservation.

## Transplantation of cryopreserved spermatogonia and cryopreservation/grafting of immature testicular tissue

Cytotoxic treatments have been observed to inflict harm to the male gonads. The testes have a considerable low tolerance for radiation, and even exposure to small doses can have gonadotoxic implications. In the case of pubertal males, semen can be recovered before the initiation of gonadotoxic therapy, thereby allowing the cryopreservation of sperm for future use. The method of intracytoplasmic sperm injection (ICSI) has proven to be successful even when the number of cryopreserved sperm is limited [98, 99]. However, prepubertal boys present a significant challenge to fertility preservation due to their inability to produce mature sperm for cryopreservation. Differentiating spermatogonia have a high rate of proliferation, rendering them highly vulnerable to cytotoxic agents [100]. Consequently, while the prepubertal testis does not complete spermatogenesis, scientific evidence suggests that such treatments can have an impact on the future fertility of prepubertal boys [101–103]. The recovery of sperm production after gonadotoxic treatment is dependent on the survival and ability of mitotically quiescent spermatogonial stem cells (SSCs) (type A dark), which should transform into actively proliferating cells and differentiate into spermatogonia (type A pale) [104].

Over the past 30 years, a variety of methodologies have surfaced, which have expanded the available treatment alternatives for males who are infertile due to non-production of sperm. These methodologies include, but are not limited to, spermatogonial stem cell (SSC) transplantation, SSC culture, testicular tissue graft, testicular tissue culture, stem cell induction, genome sequencing, and precision medicine, as well as gene therapy. Nonetheless, the majority of these novel options are still in the investigative and developmental stages and solely accessible within an experimental context. Since prepubertal testicular tissue harbors SSCs, these cells can be cryopreserved either in cell suspension [105] or directly in the tissue [106–108]. Furthermore, it is worth noting that in 20% of Tanner stage II boys, spermiation has already commenced [109], thus enabling the cryopreservation of sperm in these instances.

Currently, there exists a dearth of information regarding the optimal cryoprotectant for preserving human testicular cells with minimal damage. Nonetheless, dimethyl sulfoxide (DMSO) has emerged as the predominant choice in samples taken from prepubertal boys [110, 111]. To date, only two trials have been conducted to evaluate the viability of immature testicular tissue following vitrification. These trials have demonstrated that a low concentration of cryoprotectant can mitigate organelle and cell membrane damage, leading to improved sperm survival rates [112]. Currently, numerous strategies for storing SSCs are being actively explored.

### Testicular cell suspension

The suspension of testicular cells involves the mechanical and/or enzymatic breakdown of testicular tissue, which has an impact on cell survival and cell-to-cell interactions that are essential for cell proliferation and differentiation, as highlighted by Brook et al. [105] and Griswold et al. [113]. Studies conducted on numerous animal models have revealed that post-thaw cell viability ranges from 29 to 82%, as noted by Geens et al. [114]. Although there are fewer studies conducted on humans, Brook et al. [105], Pacchiarotti et al. [115], Unni et al. [116], and Sa et al. [117] have reported up to 60% viability. However, only Yango et al. [118] have demonstrated that the viability of fetal SSCs is comparable in both cell suspension cryopreservation and testicular tissue. Consequently, the primary disadvantages of this approach are the loss of seminiferous tubule integrity due to enzymatic digestion of the tissue and the loss of SSCs during their extraction.

### Cryopreservation of immature testicular tissue

The process of cryopreserving immature testicular tissue involves obtaining tissue through an open biopsy prior to gonadotoxic therapy, followed by cutting tissue fragments between 1 and 25 mm^3^ for cryopreservation through a slow freezing method*.* Despite the invasive nature of testicular biopsy in young patients, it is generally considered safe with no long-term impact on testicular anatomy, growth, or hormonal function, as evidenced by several studies [107, 110, 119–122]. Minimal adverse effects have been observed up to 12 months after surgery [120]. Currently, there are three protocols available for cryopreservation of immature human testicular tissue that utilize different cryoprotective agents, including ethylene glycol, HSA, sucrose, and DMSO, for slow freezing [106–108, 123]. It is worth to mention that fetal and prepubertal testicular tissue retain their structural integrity and functional capacity after cryopreservation, making testicular tissue cryobanks advantageous over suspension SSCs in preserving cellular interactions, epithelial barriers, extracellular matrix, and basal membrane [106, 107]. However, the assessment of reproductive potential after thawing requires further validation, and current strategies are considered experimental. Furthermore, none of the protocols used for freezing testicular tissue in boys and prepubertals has proven to be more efficient than others.

The techniques of spermatogonial stem cell transplantation and testicular tissue grafting have facilitated the generation of sperm and embryos or offspring across various mammalian species, including non-human primates, as demonstrated by several studies [124–133]. However, it is important to note that in oncological conditions, particularly in hematological cancers like leukemias, there is a potential risk of reintroducing malignant cells, since the testicles can serve as reservoirs for such cells. Studies in mice have shown that even transplanting as few as 20 leukemic cells can result in malignant relapse [134]. Therefore, it is crucial to ensure that there is no infiltration of tumor cells in SSC transplantations at the clinical stage, which can only be achieved by eliminating malignant cell contamination from testicular cell suspensions. Various methodologies have been proposed by different groups to remove malignant cell contamination from such suspensions, such as using flow cytometry to separate CD45-negative SSCs, as observed in mice [135–137]. Hematological cancers, particularly acute leukemias, are more common among children, highlighting the need for developing appropriate technologies to obtain mature sperm in vitro from SSCs to address this issue.

### Restoration of spermatogonial function

The seminal work by Brinster and Zimmermann [138] represents the pioneering successful transplantation of spermatogonial stem cells (SSCs) into the seminiferous tubules of mice that had undergone busulfan treatment to eradicate endogenous spermatogenesis. Since then, various methodologies have been developed to restore fertility in several mammalian species. For instance, Hermann et al. [130] reported the recuperation of spermatogenesis following transplantation of autologous or allogeneic SSCs in non-human primates rendered infertile by gonadotoxic therapy. Currently, diverse ongoing investigations in infertile animal models are being conducted, wherein SSCs are being transplanted to obtain mature and fertilization-competent sperm. While autotransplantation remains the most widely accepted method, allogeneic or xenotransplantation has been successfully employed in mice, dogs, farm animals, and macaques [124, 139–141]. In humans, only one study has reported on autologous frozen-thawed testicular cell transplantation following gonadotoxic treatment, wherein seven patients had their SSCs re-injected into their testicles post completion of the antitumor treatment. However, no follow-up data have been published for these patients [142].

The cultivation of in vitro SSCs in mice was first conducted by Kanatsu-Shinohara et al. [143]. These in vitro-grown SSCs exhibited the ability to restore spermatogenesis in infertile mice and produced competent spermatozoa capable of fertilization and successful production of offspring [143]. This culture system served as a foundation for rat and human SSCs. Sadri-Ardekani et al. [144, 145] were the first to report on the culture of adult SSCs and long-term human prepubertal. Numerous investigations have since been published in which human SSCs were cultivated following the Kanatsu-Shinohara model. However, not all reports indicate a substantial expansion of spermatogonia during the culture period. Some studies report a decrease during the culture period [146–149]. Therefore, further investigation is necessary to identify appropriate requirements for achieving successful conditions for in vitro proliferation of SSCs.

Since 2002, successful results have been obtained through the ectopic and orthotopic transplantation of testicular tissue from a variety of species, including mice, goats, and pigs, as reported by Honaramooz et al. [150] and Schlatt et al. [131]. On the other hand, Luetjens et al. [151] have reported the recovery of spermatogenesis through the autotransplantation of prepubertal testicular tissue from non-human primates, whether fresh or cryopreserved, into the scrotum.

Regarding xenotransplantation, prepubertal mouse testicular tissue has been successfully transplanted into rats, pigs, goats, and non-human primates, resulting in the production of viable sperm for fertilization [128]. Similar experiments involving the transplantation of human testicular tissue into mice did not yield complete spermatogenesis, possibly due to phylogenetic disparities between the two species [123]. As previously mentioned, it should be noted that the autotransplantation of cryopreserved testicular cells or tissues carries the risk of reintroducing malignant cells, particularly in patients with hematological or testicular cancers. As such, the methodologies of testicular cell/tissue xenotransplantation and testicular tissue culture are currently being developed and studied, with promising results.

Animal studies have exhibited the practicability and safety of reproductive technologies which employ frozen and thawed testicular tissues. However, to date, there has been no documentation of live human births resulting from the utilization of these technologies. Therefore, it is recommended that cryopreservation of immature testicular tissue be deemed experimental and offered exclusively to prepubertal patients who face significant infertility risks due to their medical condition or treatment, and only as part of a clinical trial. Prepubertal boys lack the option to preserve a semen sample prior to gonadotoxic therapy; however, their testes contain A dark and A pale SSC [152], which permits the initiation of spermatogenesis during puberty. Numerous centers across the globe, including the USA, undertake cryopreservation of testicular tissue or cells in anticipation and with the expectation that experimental therapies based on SSCs will become available shortly [107, 110, 119, 121, 145, 153].

## Testicular organoids (TOs)

Gonadotoxic therapies have the potential to obliterate not only germ cells but also the somatic layer, specifically Leydig and Sertoli cells [154, 155]. In recent years, testicular organoids (TOs) have been fabricated from isolated cells derived from immature testicular tissue for future transplantation into patients. This innovative approach has the potential to conserve and reinstate fertility in cancer patients. Matrigel and collagen were among the different types of cell matrices tested in 3D cultures of mice and rats. The resulting organoids demonstrated a comparable structure and functionality to those observed in vivo [156–160]. Vermeulen et al. [161] created TOs using hydrogels derived from decellularized immature porcine testicular tissue, observing the assembly of Sertoli cells and germ cells into seminiferous tubule-like structures that were contained by a basement membrane. Leydig cells (LCs) and peritubular cells were located outside of the tubules. Furthermore, the culture was maintained for 45 days, and the secretion of stem cell factor and testosterone was observed. Baert et al. [162] generated organotypic testicular tissue (OTs) from adult patients and a pubertal patient (15 years old) using a human decellularized testicular matrix as the scaffold. Although the OTs exhibited functionality throughout the culture, they did not demonstrate the typical tissue architecture of the testicle. This could be attributed to the degradation of the scaffold matrix by enzymes secreted by the cells.

To date, the available 3D farming systems only enable short-term follow-ups [163, 164]. Further research in this area is essential because it may provide a useful fertility preservation strategy for prepubertal or adult patients with non-obstructive azoospermia.

## Conclusions

The present state of methods currently in existence and undergoing experimental development for the preservation of fertility in boys and girls who have been diagnosed with cancer has been briefly reviewed. The awareness of potential avenues for preserving fertility in the pediatric population must be considered by the various stakeholders in healthcare in order to furnish patients facing cancer with comprehensive and precise information.

In 2019, the American Society for Reproductive Medicine (ASRM) determined that ovarian tissue banking is a permissible technique for fertility preservation and is no longer deemed experimental. This decision was based on the safety of ovarian tissue procurement for patients and the effectiveness of both tissue cryopreservation and orthotopic transplantation. However, certain aspects, such as the standardization of surgical techniques for procurement and subsequent transplantation, as well as the limited live birth rate reported to date, require clarification. Consequently, ovarian tissue banking is not the preferred fertility preservation strategy in pubertal patients when compared to oocyte cryopreservation. Nonetheless, it is crucial to evaluate each patient, regardless of their pre-, peri-, or pubertal status comprehensively. This includes assessments of pathology, treatment, sexual and psychological maturity, and potential damage to fertility. This practice will enable the medical team to recommend the most appropriate option.

For pubertal male patients, sperm cryopreservation is the recommended option. However, in prepubertal boys, the situation is still quite complex since fertility preservation strategies are still experimental, and scientific evidence is lacking. Some clinics in the USA and other parts of the world offer freezing of testicular tissue or cells as a backup option in case strategies for obtaining mature sperm from testicular tissue become available in the near future [107, 110, 119, 121, 145, 153].

## Data Availability

No new data were generated or analyzed in support of this research.
